# A Novel Mechanism of the c-Myc/NEAT1 Axis Mediating Colorectal Cancer Cell Response to Photodynamic Therapy Treatment

**DOI:** 10.3389/fonc.2021.652831

**Published:** 2021-07-28

**Authors:** Kuijie Liu, Sanlin Lei, Yong Kuang, Qianqian Jin, Dongju Long, Chao Liu, Yuhong Jiang, Hua Zhao, Hongliang Yao

**Affiliations:** Department of Gastroenterology, The Second Xiangya Hospital, Central South University, Changsha, China

**Keywords:** colorectal cancer, photodynamic therapy (PDT), NEAT1, c-Myc, the miR-124/iASPP/p53 feedback loop

## Abstract

Photodynamic therapy (PDT) is considered a potential treatment regimen for colorectal cancer cases (CRC). p53 signaling and the miR-124/iASPP axis play an essential role in the PDT resistance of CRC cells. PDT treatment downregulated NEAT1 expression in p53^wt^ HCT116 and RKO cells. In these two cell lines, NEAT1 silencing enhanced the suppressive effects of PDT on cell viability and apoptosis. Within the subcutaneously implanted tumor model, NEAT1 silencing enhanced PDT-induced suppression on tumor growth. Regarding p53-deleted HCT116 cells, PDT only moderately affected cell proliferation but induced downregulation of NEAT1. NEAT1 directly targeted miR-124, acting as a ceRNA, competing with iASPP for miR-124 binding, and counteracting miR-124–mediated repression on iASPP under PDT treatment. NEAT1 silencing was enhanced, whereas miR-124 inhibition attenuated PDT effects on CRC cells; miR-124 inhibition significantly reversed the roles of NEAT1 silencing in PDT-treated CRC cells. miR-124 negatively correlated with NEAT1 and iASPP, respectively, whereas NEAT1 and iASPP positively correlated with each other. PDT downregulated c-Myc in CRC cells, and c-Myc activated the transcription of NEAT1 through the targeting of its promoter region. Within p53^mut^ SW480 cells, PDT failed to alter cell viability and apoptosis but still downregulated c-Myc, NEAT1, and iASPP and upregulated miR-124. In p53 mutant high-abundant CRC tissues, c-Myc and NEAT1 were up-regulated, and miR-124 was downregulated. In c-Myc high-abundant CRC tissues, NEAT1 and iASPP were up-regulated, and miR-124 was downregulated. The critical role of the c-Myc/NEAT1 axis in mediating CRC response to PDT treatment *via* the miR-124/iASPP/p53 feedback loop was conclusively demonstrated.

## Introduction

Colorectal cancer is the most common digestive tract malignancy that originates from the colorectal mucosa. The morbidity and fatality associated with colorectal cancer have sharply risen worldwide in recent years (1, 2). Late clinical diagnoses in the middle and advanced stages deny the patients of their best window for successful tumor treatment through the trifecta of surgery, chemotherapy, and radiotherapy. The low early diagnosis rate has a consequential impact on the 5-year survival rate of CRC patients, which seriously affects their physical and mental health (3). The exploration of novel and combined treatment is a matter of utmost concern and urgency.

Photodynamic therapy (PDT) is a type of light-excited chemotherapy, whereby an excited photosensitizer induces the synthesis of radical oxygen species (ROS), causing oxidative injury to adjacent cells, finally leading to cell death (4). Since PDT was listed as the primary therapy for tumor treatment in 1997, the single utilization of PDT or its combination with surgery, radiotherapy, and chemotherapy was extensively employed in the treatment of malignancies (5–7). Despite the promising results yielded by PDT in advanced CRC cases (8–10), significant hurdles are brought about by PDT resistance. Previous reports indicate that the miR-124/iASPP/p53 feedback loop exerted a substantial effect on PDT resistance of CRC cells (11). In regular CRC cells, p53 targets the miR-124 promoter to enhance the expression of miR-124 and to inhibit miR-124 downstream target iASPP expression, therefore, enhancing the suppressive role of PDT in the regular proliferation of CRC cells. The mutation or deletion of p53 attenuated the binding between p53 and miR-124, which resulted in the iASPP upregulation and the enhanced proliferation of CRC cells in response to PDT (11). Considering that miR-124/iASPP/p53 feedback loop played a vital role in the PDT resistance of CRC cells, its related mechanisms were subsequently studied.

The advent of high throughput technology led to the discovery of an increasing number of CRC-related long non-coding RNAs (lncRNAs) and miRNAs (8–11). It has been demonstrated that these RNAs contribute to various aspects of carcinogenesis, such as PDT resistance through multiple mechanisms. As mentioned above, miR-124 exerts a tumor suppressor effect on CRC through binding to iASPP and promoting p53 expression (11). In another study undertaken by our group, the non-coding RNAs and mRNAs differentially expressed between CRC cells with or without PDT treatment were identified through whole transcriptome resequencing. Furthermore, it was demonstrated that the lncRNA LIFR-AS1/miR-29a/TNFAIP3 axis had a critical role in the PDT resistance of CRC cells (12). Interestingly, in addition to lncRNA LIFR-AS1, the other lncRNA, NEAT1, was also significantly downregulated by PDT treatment. NEAT1 is well known for its oncogenic role in CRC. Through interaction with different miRNAs and signaling pathways, NEAT1 promotes the proliferation, migration, invasion, and resistance to the 5-FU treatment of CRC cells (13–16). NEAT1 was found significantly correlated with miR-124, iASPP, and p53, respectively; through whole transcriptome resequencing data, NEAT1 is involved in the PDT resistance of CRC cells in a miR-124/iASPP/p53 feedback loop would constitute a logical hypothesis.

Therefore, the NEAT1 expression within CRC cells upon PDT treatment, the roles of NEAT1 in PDT-treated or non-treated CRC cell proliferation, and the effects of NEAT1 on the tumor growth of subcutaneously implanted tumor in model mice with or without PDT treatment were investigated in this study. p53 was overexpressed or deleted in p53^wt^ HCT116 cells, and the roles of PDT in cell proliferation and NEAT1 expression were determined. The predicted NEAT1 binding to miR-124 was verified using RIP and dual-luciferase reporter assays, the dynamic effects of NEAT1 and miR-124 on p53^wt^ HCT116 cell proliferation under PDT treatment were examined. In tissue samples, the expression and correlation of NEAT1, miR-124, and iASPP were studied and analyzed. Next, considering that PDT could affect NEAT1 expression in p53-deleted cells, the expression of transcription factors, FoxO3, Nrf2, and c-Myc in response to PDT treatment was determined. The binding between c-Myc and the NEAT1 promoter region was verified. The effects of PDT on cell proliferation and the expression levels of c-Myc, NEAT1, miR-124, and iASPP were subsequently examined within the p53^wt^ RKO cell line and p53^mut^ SW480 cell line. Finally, CRC tissues were examined for p53 mutant abundance, and the levels of PCNA, c-Myc, NEAT1, and miR-124 were determined in tissues with different p53 abundance. CRC tissues were examined for c-Myc abundance, NEAT1, miR-124, and iASPP expression in tissues with different c-Myc abundance was determined.

## Materials and Methods

### Clinical Samples Collection

A total of 12 cases of CRC tissues and 12 paired adjacent normal tissues were harvested through surgical resection from patients diagnosed with CRC at the Second Xiangya Hospital. This study was sanctioned by the ethics committee of the Second Xiangya Hospital [2018(Yan149)], and informed consent was obtained from each patient enrolled. All harvested tissue samples were frozen at −80°C immediately or fixed in formalin for further experiments.

### Cell Resource and Cell Culture

A normal human colon epithelial cell line, NCM460, was obtained from Lonza (Basel, Switzerland) and cultured in DMEM: F12 medium (Gibco, Waltham, MA, USA) supplemented with 10% FBS (Invitrogen, Waltham, MA, USA). Four human colorectal cancer cell lines, HCT116 (p53^wt^; CCL-247™), RKO (p53^wt^; CRL-2577™), LoVo (p53^wt^; CCL-229™), and SW480 (p53^mut^; CCL-228™), were procured from ATCC (Manassas, VA, USA). HCT116 cells were cultured in McCoy’s 5a medium modified (Gibco) supplemented with 10% FBS (Invitrogen, CA, USA). RKO cells were cultured in an Eagle’s Minimum Essential Medium (Gibco) supplemented with 10% FBS (Invitrogen). LoVo cells were cultured in F-12K medium (ATCC) supplemented with 10% FBS (Invitrogen). SW480 cells were cultured in Leibovitz’s L-15 medium (ATCC) supplemented with 10% FBS (Invitrogen). All cells were cultured at 37°C in 5% CO_2_.

### Cell Transfection

Agomir-124 or antagomir-124 was synthesized and obtained from GenePharma (Shanghai, China) for miR-124 overexpression or inhibition; agomir-NC or antagomir-NC was used as a negative control (GenePharma). For p53 or c-Myc overexpression, pcDNA3.1-p53-overexpressing vector (p53 OE) or pcDNA3.1-c-Myc (c-Myc) were used. The pcDNA3.1 (vector) was used as a negative control. All the vectors were purchased from Addgene (MA, USA). For NEAT1 or c-Myc silencing, the small interfering RNA targeting NEAT1 or c-Myc was synthesized and obtained from GenePharma; si-NC was used as a negative control. These vectors (1 µg/ml) and siRNAs (50 nM) were subsequently transfected in target cells with Lipofectamine 3000 Reagent (Thermo Fisher Scientific, Waltham, MA, USA) as per established protocols. The primer for vector construction and the sequence of siRNAs were listed in **Table S1**.

### RNA Extraction and SYBR Green Quantitative PCR Analysis

The TRIzol reagent (Invitrogen, CA, USA) was used to extract the total RNA target tissues or cells. The expression levels of lncRNA, miRNA, and mRNAs were determined using a SYBR Green qPCR assay (Takara, Dalian, China) accordingly to the manufacturer’s instruction. The expression of RNU6B (for miRNA) or GAPDH (for mRNA) served as an endogenous control. The data were processed according to the 2^−ΔΔCT^ method. The primers were listed in **Table S1**.

### Photodynamic Therapy (PDT) and Photo-Toxicity Experiments

5-aminolevulinic acid (5-ALA) (Sigma-Aldrich, Shanghai, China) was used as photosensitizers in this study. CRC cells were incubated with 0, 5, 10, or 20 mg/L 5-ALA for 6 h and then subjected to PDT experiments. Photosensitized cells were briefly irradiated with visible light from LD-630 laser tumor therapeutic equipment (Leimai Tech, Shenzhen, China). The average fluence rate was 15 mW/cm^2^. A light dose of 60 J/cm^2^ was used. The PDT-treated or non-treated CRC cells were then examined for cell viability 24 h after photodynamic therapy, cell apoptosis, NEAT1 expression levels, and so on, were examined as well.

### CCK-8 for Cell Viability

Cell viability was determined using a CCK-8 kit (Beyotime, Shanghai, China) as per the manufacturer’s instructions. In general, 5 × 10^3^ cells were seeded in each well in a 96-well plate for 24 h, transfected or non-transfected for 48 h, subjected or non-subjected to PDT treatment, 24 h later cells were incubated with a CCK-8 reagent (10 μl) for 1 h. The background control wells without cells were also added CCK-8 reagent for 1 h. At the end of the incubation, the OD450 value was determined for each well using a microplate reader (Bio-rad, CA, USA). The average absorbance of the control wells was subtracted from that of the other wells.

### Flow Cytometry for Cell Apoptosis

The cell apoptosis rate was examined by Annexin V-FITC Apoptosis Detection Kit (Cat. KGA104, Keygen, Nanjing, China). In general, targeted cells were collected with 0.25% trypsin without EDTA. After washing twice with ice-cold PBS, cells were re-suspended in 500 μl of binding buffer. The cells were incubated with a 5-μl antibody against Annexin V-FITC and 5-μl propidium iodide (PI) for 15 to 20 min in the dark. At the end of the incubation, Novocyte flow cytometer (Anglient, USA) was used to detect the cell apoptosis with an excitation wavelength (Ex) of 488 nm and emission wavelength (Em) of 530 nm. 10,000 cells were analyzed in each group.

### Subcutaneously Implanted Tumor Model in Nude Mice

Female BALB/c nude mice (5-week-old; Hunan SJA Laboratory Animal Co., Ltd, Changsha, China) were used to establish the subcutaneously implanted tumor model. All procedures were sanctioned by the ethics committee of the Second Xiangya Hospital of Central South University (2018(Yan149)). HCT116 and RKO cells, infection with lentivirus contained si-NC or si-NEAT1 (Genechem, Shanghai, China), were used for the mice injection. Cells (5 × 10^5^) were suspended in 100 μl of serum-free DMEM mixed with 100 μl of 10% Matrigel (BD, NJ, USA) and injected subcutaneously to each mouse.

From day 7 of subcutaneous injection, mice in the treatment group received an intraperitoneal injection of 250 mg/kg of 5-ALA (17, 18). Five hours following injection, mice in the treatment group were exposed to visible light from LD-630 Laser tumor therapeutic equipment (Leimai Tech) at a measured fluence rate of 96 mW/cm^2^ and fluence of 32 J/cm^2^. Mice in the control group were unexposed to irradiation. Three weeks following the initial treatment (day 27 of subcutaneous injection), the mice were anesthetized and sacrificed. Tumor volume, tumor weight, and the protein levels of PCNA, Caspase3, and cleaved-Caspase3 in tumor tissues were examined. The methods were carried out in accordance with the relevant guidelines and regulations.

### Immunoblotting

The total protein was extracted from target tissues or cells, using a radioimmunoprecipitation assay (RIPA) lysis buffer (Beyotime, Shanghai, China) with PMSF (Beyotime). The protein concentration was determined using the BCA protein assay kit (Beyotime). The protein was subsequently separated using 8% to 15% SDS-PAGE electrophoresis, and the proteins were wet electro-transferred to PVDF membranes. Prior to incubation with proper primary antibodies, the membranes were blocked with a 5% milk solution for 1 h to prevent nonspecific bindings. The following primary antibodies were used to incubate the membranes overnight at 4°C: anti-PCNA (dilution 1:1000, 10205-2-AP; Proteintech, Wuhan, China), anti-Caspase 3 (dilution 1:1000, 19677-1-AP, Proteintech), anti-cleaved-Caspase 3 (dilution 1:1000, ab2302; Abcam, Cambridge, MA, USA), anti-p53 (dilution 1:1000, CSB-PA07889A0Rb; Cusabio, Wuhan, China), anti-iASPP (dilution 1:1000, ab115605, Abcam), anti-c-Myc (dilution 1:1000, 10828-1-AP; Proteintech), and anti-GAPDH (dilution 1:2000, 60004-1-Ig; Proteintech). The membranes were then incubated with secondary antibodies (dilution 1:5000, SA00001-1 and SA00001-2, HRP-labeled goat anti-rabbit IgG and goat anti-mouse IgG; Proteintech) at room temperature for 1 h and washed thrice with 0.05% TBST for 5 min each time. The signal was detected by the enhanced chemiluminescence (ECL) kit (Beyotime). The blot bands were observed by an automatic chemiluminescence imaging system (Tannon, Shanghai, China).

### Dual-Luciferase Reporter Assay

For lncRNA NEAT1 binding miR-124, lncRNA NEAT1 sequence containing the miR-124 binding sites was fused into psicheck2 luciferase reporter vector (Promega) to generate wild-type reporter vector (wt-lncNEAT1); meanwhile, site-directed mutagenesis of the miR-124 binding sites in lncRNA NEAT1 sequence was introduced into luciferase reporter vector to generate a mutant-type reporter vector (mut-lncNEAT1). 293T cells were subsequently co-transfected with agomir-124/antagomir-124 with these reporter vectors using Lipofectamine 3000. The luciferase activity was determined 24 h post-transfection using a Dual-Luciferase Reporter System (Promega) as per the manufacturer’s instructions.

For c-Myc binding lncRNA NEAT1 promoter region, NEAT1 promoter region containing c-Myc response element (c-Myc RE) was fused into psicheck2 luciferase reporter vector (Promega) to generate wild-type reporter vector (psiCheck2-proNEAT1); similarly, mutagenesis of the c-Myc RE in lncRNA NEAT1 promoter region was introduced into the luciferase reporter vector to generate the mutant-type reporter vector (psiCheck2-proNEAT1-mut). 293T cells were then co-transfected with pcDNA3.1-c-Myc together with these reporter vectors using Lipofectamine 3000. The luciferase activity was determined 24 h post-transfection using Dual-Luciferase Reporter System (Promega) as directed by the manufacturer’s instruction. The primers for plasmid construction are shown in **Table S1**.

### RNA Immunoprecipitation (RIP) Assay

A Magna RIP kit (Millipore, Bedford, MA, USA) was used to perform the RIP assay. HCT116 cells were washed by cold PBS and scraped into a tube. After centrifugation (15,000 rpm × 5 min at 4°C), cells were lysed by lysis buffer containing a protease inhibitor cocktail and RNase inhibitor. The magnetic beads were suspended in 100 µl RIP washing buffer and incubated with 5 μg human anti-Ago2 (ab32381; Abcam) or anti-IgG (negative control) for 30 min in room temperature. After discarded the suspension, the Ago2- or IgG-beads magnetic beads complex were re-suspended in 900 μl RIP immunoprecipitation buffer and incubated with 100 μl cell lysis supernatant overnight at 4°C with rotation. At the end of the incubation, the RNA-Ago2 or IgG beads magnetic beads were washed six times with RIP washing buffer and then suspended in 150 μl protein K buffer for 30 min at 55°C. Next, the supernatants were transferred to a tube and underwent RNA purify procedure. Briefly, the RNA in the supernatants was isolated by Trizol and chloroform and precipitated by ethanol. Finally, the purified RNA has undergone RT-PCR to determine the levels of miR-124 and lncRNA NEAT1. The primers were listed in **Table S1**.

### Chromatin Immunoprecipitation Assay

Chromatin immunoprecipitation (ChIP) assays were performed using the EZ-ChIP kit (Millipore) using the previously employed methods (11). Briefly, HCT116 cells growth in the dish were washed by cold PBS and then treated with formaldehyde and gently swirled the dish to mix for 10 min. The dish was added with 2-ml PBS containing protease inhibitor cocktail and scraped cells to a tube. After centrifugation (700*g* for 5 min at 4°C) and discarding the supernatant, the cell pellet was re-suspended in SDS lysis buffer and sonicated on ice. The cell lysate was centrifuged at 10,000*g* for 10 min at 4°C and removed the supernatant to a fresh tube. Then, the tube was added with dilution buffer and Protein G agarose for 1 h at 4°C with rotation. The supernatant was removed to a fresh tube and incubated with 1 μg anti-c-Myc (18583, CST, USA) or anti-IgG (negative control) overnight at 4°C with rotation. Then, the supernatant-antibody complex was incubated with 60 μl Protein G agarose for 1 h at 4°C with rotation. The Protein G agarose-antibody/chromatin complex was collected by centrifugation and eluted by elution buffer. The supernatant was removed to a fresh tube to undergo DNA-protein crosslinks reverse. Finally, the DNA was precipitated by Bind Reagent A and eluted by Elution Buffer C according to the manufacturer’s instruction. The purified DNA has undergone qRT-PCR to determine the levels of NEAT1 promoter. The ChIP-PCR primers were designed to amplify the promoter regions containing putative c-Myc-binding sites within the lncRNA NEAT1 promoter region. The primers were listed in **Table S1**. The relative promoter abundance was calculated as the ratio of the amplification efficiency of the ChIP sample to that of the non-immune IgG.

### H&E Staining

Tissue samples were fixed in 4% paraformaldehyde, embedded in paraffin, and cut into 4-μm-thick cross-sections. H&E staining was performed for the observation of histopathological features (19). A minimum of five fields was analyzed on each section, and representative images were subsequently captured by an Olympus microscope (CKX53, Olympus, Japan).

### Immunohistochemical Staining

Formalin-fixed paraffin-embedded human tissue specimens were sliced into 4- μm-thick cross-sections. The tissue sections were permeabilized with 0.2% triton (Sigma, St. Louis, MO, USA) at room temperature for 10 min, and then incubated with a blocking solution (3.75% BSA/5% goat serum; Zymed, Carlsbad, CA, USA) for 30 min. The samples were then incubated with an antibody recognizing human mutant forms of p53 but not human p53 wild type (ab32049; Abcam), or anti-c-Myc (10828-1-AP; Proteintech). The isotype control antibody (dilution is 1:100, ab172730; Abcam) were used as the negative control for immunohistochemical (IHC) staining. All sections were incubated with the poly-IgG-HRP antibody (SV0004; Boster, Wuhan, China) in a blocking solution for 30 min. The section was stained by a diaminobenzidine (DAB) kit (AR1022; Boster) for 10 min. The nuclei of the sections were then stained with hematoxylin (0.2 mg/ml; Sigma) and subsequently underwent microscope analysis.

Through a double-blind study undertaken by three pathologists, the mutations in all the sections were individually assessed; samples were divided into p53^mut^+ and p53^mut^++ groups following previously used methods (12). Generally, the grouping rules are as follows: 0, assigned when no staining was observed; 1, when <10% of tumor cell nuclei were reactive; 2, when >10%, but <33% of the nuclei stained; and 3, if >33% of nuclei were positive (20); 0–2 was defined as p53^mut^+, and 3 was defined as p53^mut^++. A similar evaluation was performed to divide the samples into c-Myc + and c-Myc ++ groups.

### Statistics Analysis

The results of at least three independent experiments were processed using GraphPad (San Diego, California, USA) and expressed as mean ± standard deviation (S.D.). A one-way analysis of variance (ANOVA) and Tukey’s multiple comparison test, or Student’s *t*-test, were used to assess its statistical significance. A *P* value < 0.05 was deemed as statistically significant.

## Results

### Silencing lncRNA NEAT1 Enhances the CRC Sensitivity to PDT

**Figure 1:**
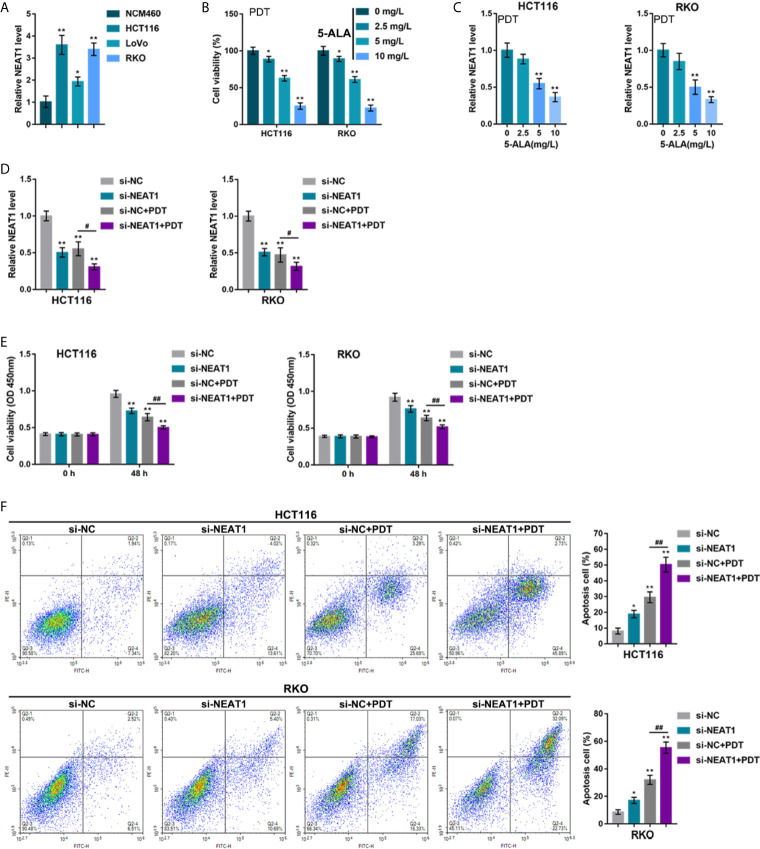
Silencing lncRNA NEAT1 enhances the sensitivity of colorectal cancer (CRC) cells to PDT treatment. **(A)** The expression levels of NEAT1 was determined in a normal cell line, NCM460, and three CRC cell lines, HCT116, RKO, and LoVo, by qRT-PCR. Then, HCT116 and RKO cells were incubated with 0, 5, 10, or 20 mg/L 5-ALA, treated with PDT and examined for cell viability by CCK-8 assay **(B)** and NEAT1 expression levels by qRT-PCR **(C)**. Next, HCT116 and RKO cells were transfected with small interfering RNA targeting NEAT1 (si-NEAT1; si-NC as a negative control), subjected to or non-subjected to PDT treatment, and examined for the expression levels of NEAT1 by qRT-PCR **(D)**, cell viability by CCK-8 assay **(E)**, and cell apoptosis by Flow cytometry **(F)**. *P < 0.05, **P < 0.01, compared with NCM460, 0 mg/L or si-NC group; ^#^ *P* < 0.05, ^##^ *P* < 0.01, compared with si-NC+PDT group.

To investigate the specific effects of NEAT1 upon PDT sensitivity of CRC cells, the NEAT1 expression within normal or CRC cells was determined. **Figure 1A** illustrates that, in comparison with that in a normal cell line, NCM460, NEAT1 expression was dramatically increased within three CRC cell lines, HCT116, RKO, and LoVo, more upregulated in HCT116 and RKO cell lines. Thus, HCT116 and RKO cell lines were incubated with 0, 5, 10, or 20 mg/L 5-ALA (photosensitizer) and treated with PDT. **Figure 1B** shows that, along with the increases in 5-ALA doses, the repressive effects of PDT on CRC cell viability were enhanced. In the meantime, PDT treatment significantly downregulated NEAT1 expression in a 5-ALA dose-dependent manner (**Figure 1C**). Thus, PDT treatment significantly inhibits CRC cell viability and NEAT1 expression.

HCT116 and RKO cell lines were subsequently transfected with small interfering RNA that targeted NEAT1 (si-NEAT1; si-NC as a negative control), subjected to or not subjected to PDT treatment, and the NEAT1 expression was determined. Within these two cell lines, NEAT1 expression was shown to be downregulated by si-NEAT1 transfection and even more downregulated by PDT treatment (**Figure 1D**). Meanwhile, NEAT1 silencing suppressed CRC cell viability and enhanced apoptosis, whereas PDT treatment enhanced the inhibited viability and enhanced apoptosis of CRC cells (**Figures 1E, F**). These data indicate that silencing NEAT1 might potentially enhance the suppressive roles of PDT in the proliferation of CRC cells.

### Silencing NEAT1 Enhances the Repressive Effects of PDT on Tumor Growth in Model Mice

**Figure 2:**
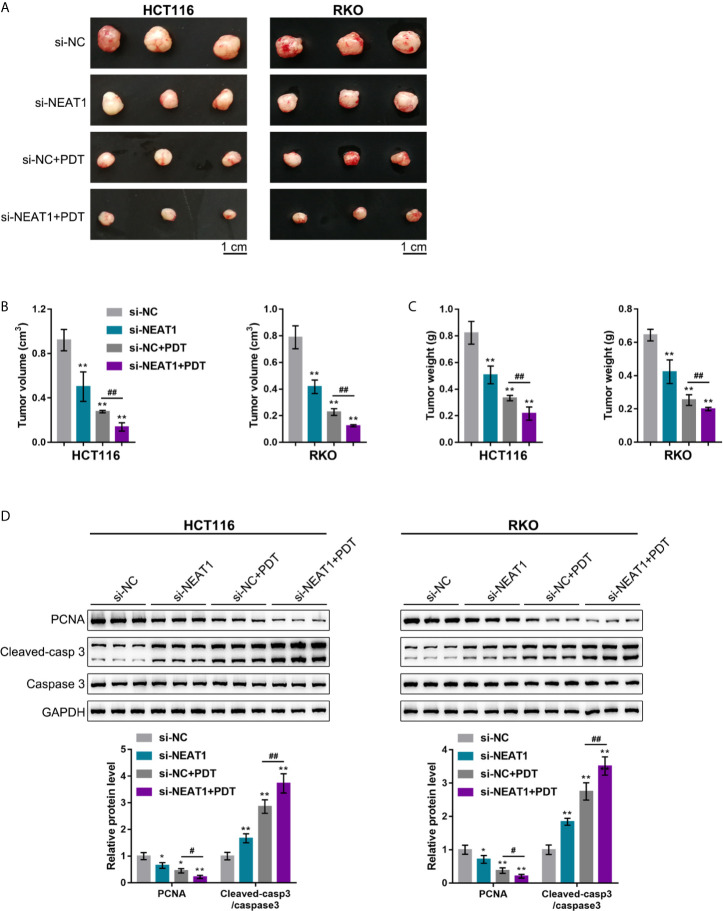
Silencing NEAT1 enhances the repressive effects of PDT on tumor growth in model mice. The subcutaneously implanted tumor model was established in nude mice by injecting HCT116 or RKO cells infected with lentivirus containing si-NC or si-NEAT1. Model mice were treated or non-treated with PDT. On day 27 of cell injection, the tumor volume and tumor weight were examined **(A–C)**, and the protein levels of PCNA, Caspase 3, and cleaved-Caspase 3 were determined in tumor tissues by Immunoblotting **(D)**. n= 6. *P < 0.05, **P < 0.01, compared with si-NC group; ^#^ *P* < 0.05, ^##^ *P* < 0.01, compared with si-NC+PDT group.

Considering the key role of cancer cell hyperproliferation in tumor growth and cancer progression, the effects of silencing NEAT1 on tumor growth upon PDT treatment were subsequently investigated. HCT116 or RKO cells infected were injected with NEAT1 knockdown lentivirus to establish the subcutaneously implanted tumor model in mice. Tumors in mice grew with or without exposure to PDT. On day 27 of cell injection, the volume and weight of the tumor, as well as PCNA, Caspase 3, and cleaved-Caspase 3 levels within tumor tissues, were examined. Silencing NEAT1 or PDT treatment alone significantly decreased the volume and weight of the tumor, and NEAT1 silencing combined with PDT treatment decreased the most (**Figures 2A–C**). Regarding proliferating and apoptotic markers, silencing NEAT1 or PDT treatment alone decreased PCNA and elevated cleaved-Caspase 3/Caspase 3 ratios, and NEAT1 silencing combined with PDT treatment further decreased PCNA and elevated cleaved-Caspase 3/Caspase 3 ratios (**Figure 2D**). These *in vivo* findings indicate that the silencing of NEAT1 enhances the suppressive roles of PDT treatment in tumor growth in the subcutaneously implanted tumor model in mice.

### NEAT1 Promoting CRC Cell Resistance to PDT Through Targeting miR-124

**Figure 3:**
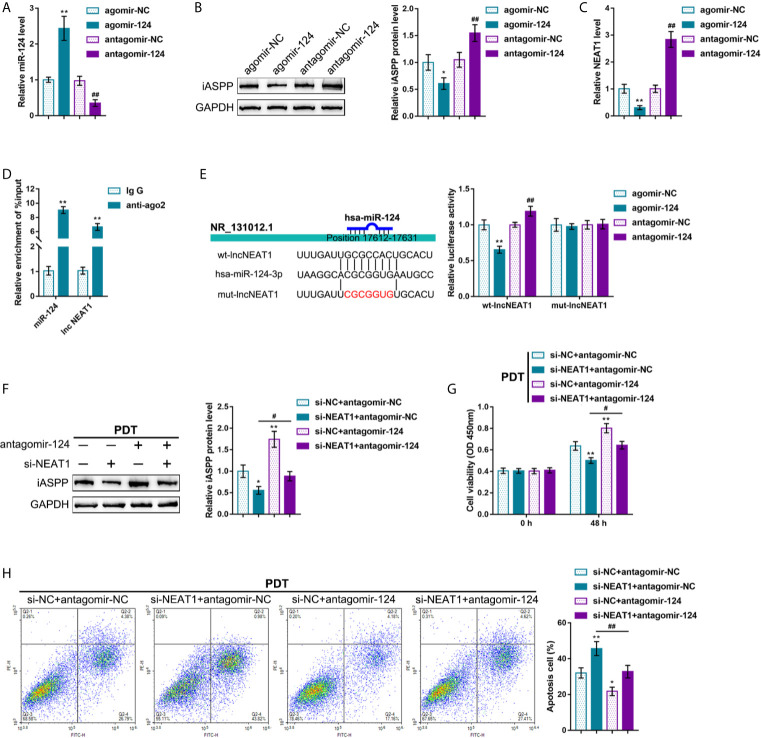
NEAT1 promoting CRC cell resistance to PDT treatment through targeting miR-124. **(A)** miR-124 overexpression or inhibition was achieved in HCT116 cells by transfecting agomir-124 or antagomir-124; agomir-NC or antagomir-NC was transfected as a negative control. The transfection efficiency was confirmed by qRT-PCR. **(B)** HCT116 cells were transfected with agomir-124 or antagomir-124 and examined for the protein levels of iASPP by Immunoblotting. **(C)** HCT116 cells were transfected with agomir-124 or antagomir-124 and examined for the levels of NEAT1 by RT-PCR. **(D)** RIP assay was performed using anti-IgG or anti-Ago2. The levels of miR-124 and NEAT1 in immunoprecipitate were determined by qRT-PCR. **(E)** Dual-luciferase reporter assay was performed by constructing wild-type and mutant-type lncNEAT1 reporter vectors and co-transfecting the reporter vectors in 293T cells with agomir-124 or antagomir-124. The luciferase activity was determined. Then, HCT116 cells were co-transfected with si-NEAT1 and antagomir-124, treated or non-treated with PDT, and examined for the protein levels of iASPP by Immunoblotting **(F)**, cell viability by CCK-8 assay **(G)**, and cell apoptosis by flow cytometry **(H)**. *P < 0.05, **P < 0.01, ^#^ *P* < 0.05, ^##^ *P* < 0.01.

It was demonstrated in our previous study that a miR-124/iASPP/p53 feedback loops. miR-124 inhibits iASPP through targeting, therefore attenuating the inhibitory effects of iASPP on p53; p53, consequently, binds to the miR-124 promoter and further activates miR-124 transcription (11). Since PDT significantly alters NEAT1 expression in either p53-overexpressed or -deleted CRC cells. Next, it was investigated whether NEAT1 affects CRC cell sensitivity to PDT through the miR-124/iASPP axis. miR-124 overexpression or inhibition in regular HCT116 cells was achieved by transfecting agomir-124 or antagomir-124; agomir-NC or antagomir-NC was transfected as a negative control, as confirmed by qRT-PCR (**Figure 3A**). In HCT116 cells, miR-124 overexpression decreased, whereas miR-124 inhibition increased the level of iASPP protein (**Figure 3B**). Moreover, miR-124 overexpression decreased, whereas miR-124 inhibition increased NEAT1 levels (**Figure 3C**). Then, using RIP and dual-luciferase reporter assays, the predicted NEAT1 binding to miR-124 was verified. First, RIP assay was performed using anti-IgG or anti-Ago2. qRT-PCR was employed to determine miR-124 and NEAT1 expression in the immunoprecipitate. **Figure 3D** depicts that miR-124 expression and NEAT1 expression were dramatically higher in anti-Ago2 immunoprecipitate compared with those in anti-IgG immunoprecipitate. Two different types of lncNEAT1 reporter vectors were subsequently constructed, wild-type and mutant-type (namely wt-lncNEAT1/mut-lncNEAT1), and were co-transfected in 293T cells with agomir-124 or antagomir-124. When co-transfected with wt-lncNEAT1, the overexpression of miR-124 was repressed, while the inhibition of miR-124 enhanced the luciferase activity; when co-transfected with mut-lncNEAT1, miR-124 overexpression or inhibition failed to alter the luciferase activity (**Figure 3E**). These data indicate that NEAT1 targets miR-124 in an Ago2-related way.

Then, HCT116 cells were co-transfected with si-NEAT1 and antagomir-124, subjected to or non-subjected to PDT treatment, and determined miR-124 downstream target iASPP protein. Under PDT treatment, NEAT1 silencing decreased, whereas miR-124 inhibition increased the iASPP protein level; the inhibitory effects of NEAT1 silencing were reversed by miR-124 inhibition (**Figure 3F**). Concerning the cellular functions, under PDT treatment, NEAT1 silencing inhibited viability and promoted apoptosis of cells, whereas the inhibition of miR-124 promoted HCT116 cell viability and inhibited cell apoptosis; miR-124 inhibition dramatically attenuated the roles of NEAT1 knockdown in CRC cells (**Figures 3G, H** **)**. These data suggest that NEAT1 affects CRC cell sensitivity to PDT through the miR-124/iASPP axis in p53^wt^ HCT116 cells.

### Expression and Correlation of NEAT1, miR-124, and iASPP Within Human CRC Tissues

**Figure 4:**
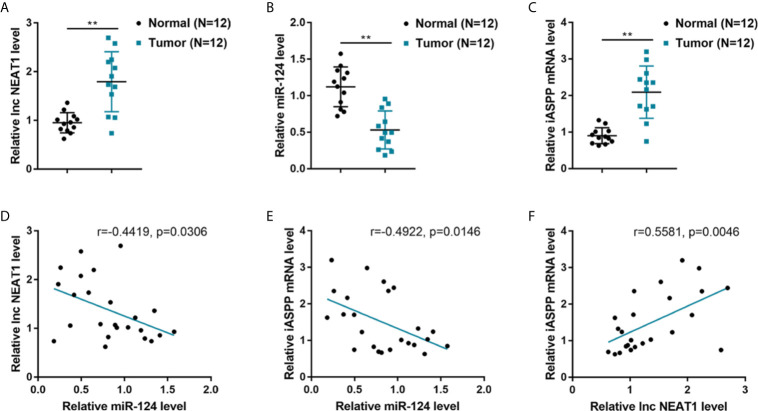
Expression and correlation of NEAT1, miR-124, and iASPP in human CRC tissue samples. **(A–C)** The expression of lncRNA NEAT1, miR-124, and iASPP were determined in 12 paired of CRC and adjacent non-cancerous tissues by qRT-PCR. **P < 0.01. **(D–F)** The correlation of lncRNA NEAT1, miR-124, and iASPP expression in tissue samples was analyzed by Pearson’s correlation analysis.

To further confirm the NEAT1/miR-124/iASPP axis within CRC, this study examined the expression and correlation of NEAT1, miR-124, and iASPP within tissue samples. lncRNA NEAT1 and iASPP levels were shown to be significantly increased, and the expression of miR-124 was shown to be decreased than that within adjacent normal control tissue samples within human CRC tissues (**Figures 4A–C**). In tissue samples, miR-124 exhibited a negative correlation with NEAT1 and iASPP, respectively, whereas NEAT1 was positively correlated with iASPP (**Figures 4D–F**). It is, therefore, concluded that deregulation of NEAT1, miR-124, and iASPP occurs in CRC tissues.

### c-Myc Activates the Transcription of NEAT1 *via* Targeting Its Promoter Region

**Figure 5:**
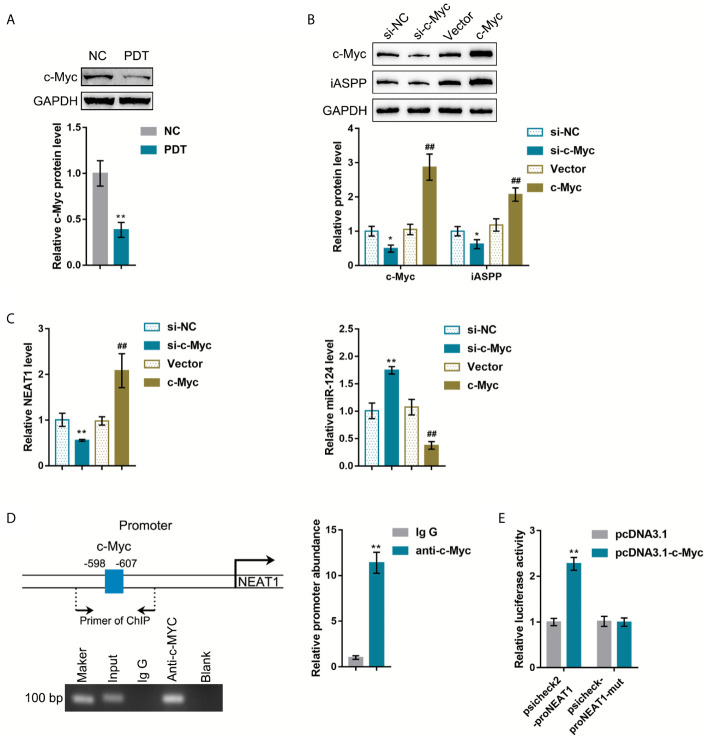
c-Myc binds to the promoter region of NEAT1 to activate the transcription. **(A)** HCT116 cells were treated or non-treated with PDT and examined for the protein levels of FoxO3, Nrf2, and c-Myc by Immunoblotting. **(B)** c-Myc overexpression or silencing was achieved in HCT116 cells by transfecting si-c-Myc or c-Myc-overexpressing vector. The protein levels of c-Myc and iASPP in transfected cells were determined by Immunoblotting. **(C)** HCT116 cells were transfected with si-c-Myc or c-Myc and examined for the expression levels of NEAT1 and miR-124 by qRT-PCR. **(D)** ChIP assay was performed using anti-c-Myc or anti-IgG. The levels of NEAT1 promoter in the immunoprecipitate were determined by qRT-PCR. **(E)** NEAT1 promoter reporter vectors, psiCheck2-proNEAT1 and psiCheck2-proNEAT1-mut, were constructed and co-transfected in 293T cells with pcDNA3.1/c-Myc. The luciferase activity was determined. *P < 0.05, **P < 0.01, ^##^ *P* < 0.01.

The oncogenic transcription factors that potentially activate NEAT1 transcription were sought to explain the abnormal regulation of NEAT1 in CRC tissues. The c-Myc transcription factor is a potent regulator of cellular proliferation and cell fate decision (21). It has been reported that PDT decreased C-Myc expression in breast cancer cells (22). ChIP-Atlas and JASPAR were used to predict whether transcription factor c-Myc targets the promoter region of NEAT1 (**Table S2**). Then, HCT116 cells were subjected to or non-subjected to PDT treatment and determined for c-Myc proteins. As illustrated in **Figure 5A**, c-Myc was significantly decreased by PDT treatment. si-c-Myc or c-Myc-overexpressing vector was, therefore, transfected to achieve c-Myc overexpression or silencing in HCT116 cells, as confirmed by immunoblotting (**Figure 5B**). Moreover, iASPP protein and miR-124 and NEAT1 expression were examined within transfected cells. As shown in **Figure 5B**, c-Myc overexpression increased, whereas c-Myc silencing decreased iASPP protein within HCT116 cells. As shown in **Figure 5C**, c-Myc overexpression upregulated NEAT1 and downregulated miR-124 expression, whereas c-Myc silencing exerted opposite effects on NEAT1 and miR-124 expression. These data indicate c-Myc positive regulation of NEAT1 and negative regulation of miR-124.

Next, ChIP and dual-luciferase reporter assays were employed to verify the binding of c-Myc to the NEAT1 promoter region. First, anti-c-Myc and anti-IgG were used to incubate HCT116 cells, and the abundance of NEAT1 promoter in the immunoprecipitate was determined using qRT-PCR. **Figure 5D** showed that NEAT1 promoter level within the anti-c-Myc immunoprecipitate was dramatically higher than that in the anti-IgG immunoprecipitate. Second, for the dual-luciferase reporter assay, NEAT1 promoter reporter vectors were constructed, psiCheck2-proNEAT1 and psiCheck2-proNEAT1-mut, and co-transfected these vectors in 293T cells with pcDNA3.1/c-Myc. As shown in **Figure 5E**, when co-transfected with psiCheck2-proNEAT1, c-Myc overexpression significantly promoted the luciferase activity. When co-transfected with psiCheck2-proNEAT1-mut, c-Myc failed to alter the luciferase activity.

### p53 Deletion Enhances the Resistance of CRC Cells to PDT

**Figure 6:**
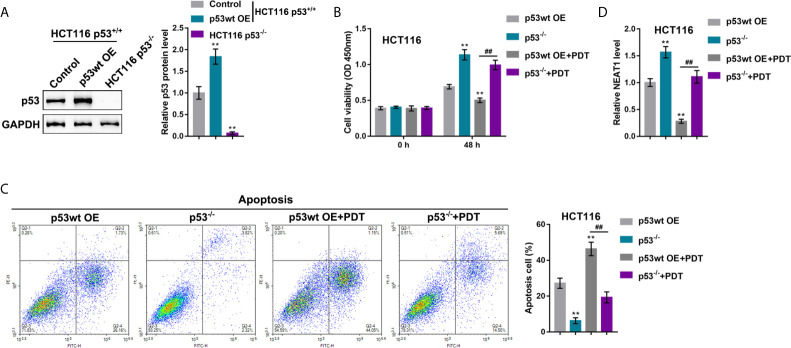
p53 deletion enhances the resistance of CRC cells to PDT. **(A)** p53 overexpression was achieved in HCT116 cells by the transfecting p53-overexpressing vector (p53 OE). The protein levels of p53 were determined in regular HCT116 cells, p53 OE-transfected HCT116 cells, and p53^−/−^ HCT116 cells by Immunoblotting. Next, p53 OE-transfected HCT116 or p53-deleted p53^−/−^ HCT116 cells were treated or non-treated with PDT and examined for cell viability by CCK-8 assay **(B)**, cell apoptosis by flow cytometry **(C)**, and the expression levels of NEAT1 by qRT-PCR **(D)**. **P < 0.01, compared to p53wt OE group; ^##^ *P* < 0.01, compared to p53wt OE+PDT group.

Our previous study demonstrated that the miR-124/iASPP axis and the p53 pathway played an important role in PDT treatment on CRC (11, 23). p53 was then overexpressed or deleted in HCT116 cells (p53^wt^) to confirm the involvement of p53 in PDT functions. p53 protein was examined within regular HCT116 cells, p53 OE-transfected HCT116 cells, and p53-deleted HCT116 cells by immunoblotting. **Figure 6A** showed that the protein level of p53 was more elevated within p53 OE-transfected HCT116 cells than that within regular HCT116 cells, and non-detected in p53-deleted HCT116 cells. Next, p53 OE-transfected HCT116 or p53-deleted p53^−/−^ HCT116 cells were subjected to or non-subjected to PDT treatment and determined for PDT effects. As for cellular functions, p53 deletion alone dramatically increased viability and inhibited apoptosis of cells, whereas PDT treatment alone on p53 overexpressed cells led to suppressed viability and enhanced apoptosis of cells (apoptosis rate was increased from 27.22% to 46.37%) within p53-deleted HCT116 cells, PDT treatment only moderately inhibited viability and enhanced apoptosis of cells (apoptosis rate was increased from 6.28% to 19.31%) (**Figures 6B, C** **)**. These data further suggest that PDT treatment on CRC cells in a way related to p53. NEAT1 expression was subsequently determined in each group. **Figure 6D** showed that the expression level of NEAT1 was significantly increased within p53-deleted HCT116 cells compared with p53-overexpressed HCT116 cells. PDT treatment decreased NEAT1 levels in both p53-overexpressed or p53-deleted HCT116 cells, and p53-overexpressed HCT116 cells showed a greater decline. In summary, in p53-deleted HCT116 cells, PDT still inhibits NEAT1 expression but fails to alter CRC cell proliferation significantly.

### p53 Mutation Enhances the Resistance of CRC Cells to PDT

**Figure 7:**
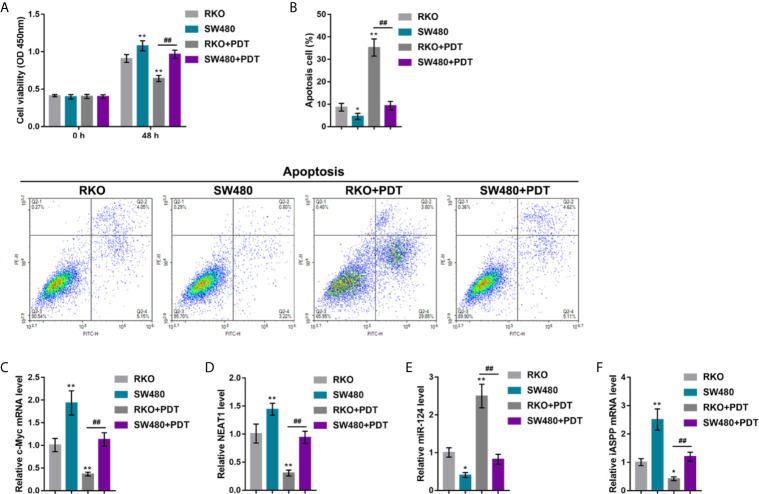
p53 mutation enhances the resistance of CRC cells to PDT. p53 wt RKO and p53 mut SW480 cells were treated or non-treated with PDT and examined for cell viability by CCK-8 assay **(A)**, cell apoptosis by Flow cytometry **(B)**, and the expression levels of c-Myc **(C)**, NEAT1 **(D)**, miR-124 **(E)**, and iASPP **(F)** by qRT-PCR. *P < 0.05, **P < 0.01, compared to RKO cells. ^##^ *P* < 0.01 compared to RKO+PDT group.

In our previous study, in p53-mutant or -deleted cells, p53 failed to bind to the miR-124 promoter, resulting in elevated expression of iASPP and promoted CRC cell viability upon PDT. The effects of p53 mutation on the c-Myc/NEAT1/miR-124/iASPP axis and CRC cell proliferation upon PDT treatment were further investigated. p53^wt^ RKO and p53^mut^ SW480 cells were treated or non-treated with PDT and examined for relevant indexes. In p53^wt^ RKO cells, PDT treatment dramatically suppressed viability and enhanced apoptosis of cells (apoptosis rate was increased from 8.66% to 35.24%); however, within p53^mut^ SW480 cells, PDT treatment only moderately altered cell viability and apoptosis (apoptosis rate increased from 4.56% to 9.34%) (*P < 0.05, **P < 0.01, **Figures 7A, B** **)**. Upon PDT treatment, the p53^mut^ SW480 cell viability was higher, and cell apoptosis was lower than that of the p53^wt^ RKO cell (##*P* < 0.01, **Figures 7A, B**). Regarding the c-Myc/NEAT1/miR-124/iASPP axis, the expression of c-Myc, NEAT1, and iASPP was higher, and the expression of miR-124 was lower within p53^mut^ SW480 cells compared with that in p53^wt^ RKO cells (*P < 0.05, **P < 0.01, **Figures 7C–F**). PDT treatment significantly upregulated miR-124 expression and downregulated c-Myc, NEAT1, and iASPP expression in p53^wt^ RKO cells than that within normal p53^wt^ RKO cells (*P < 0.05, **P < 0.01, **Figures 7C–F**). Within p53^mut^ SW480 cells treated with PDT, the expression of c-Myc, NEAT1, and iASPP was higher, and the expression of miR-124 was lower, compared with that in p53^wt^ RKO cells treated with PDT (##*P* < 0.01, **Figures 7C–F**). These data indicate that the inhibitory roles of PDT treatment in c-Myc, NEAT1, and iASPP are attenuated, leading to attenuated suppressive roles of PDT in the proliferation of p53^mut^ CRC cells.

### NEAT1/miR-124/iASPP Axis Expression Within Human CRC Tissues

**Figure 8:**
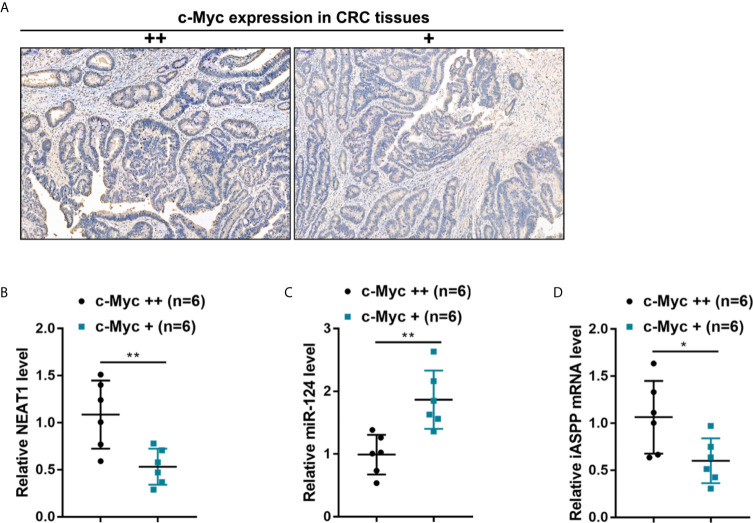
The expression of the NEAT1/miR-124/iASPP axis in human CRC tissues **(A)** By using an antibody specific to c-Myc, the c-Myc protein in CRC tissue samples was identified by IHC staining. c-Myc abundance was evaluated, and CRC tissues were divided into c-Myc+ and c-Myc++ groups accordingly. **(B–D)** The expression levels of NEAT1, miR-124, and iASPP were determined in c-Myc+ and c-Myc++ CRC tissues by qRT-PCR. *P < 0.05, **P < 0.01 compared with c-Myc+ group.

By using an antibody specific to c-Myc, c-Myc abundance was evaluated through IHC staining, and human CRC tissues were divided into c-Myc+ and c-Myc++ groups accordingly (**Figure 8A**). NEAT1, miR-124, and iASPP expressions were examined within c-Myc+ and c-Myc++ CRC tissues. As illustrated in **Figures 8B–D**, NEAT1 and iASPP expressions were higher, whereas miR-124 expression was lower in c-Myc++ CRC tissues compared with that in c-Myc+ CRC tissues.

### c-Myc/NEAT1/miR-124 Axis Expression in p53-Mutant Tissues

**Figure 9:**
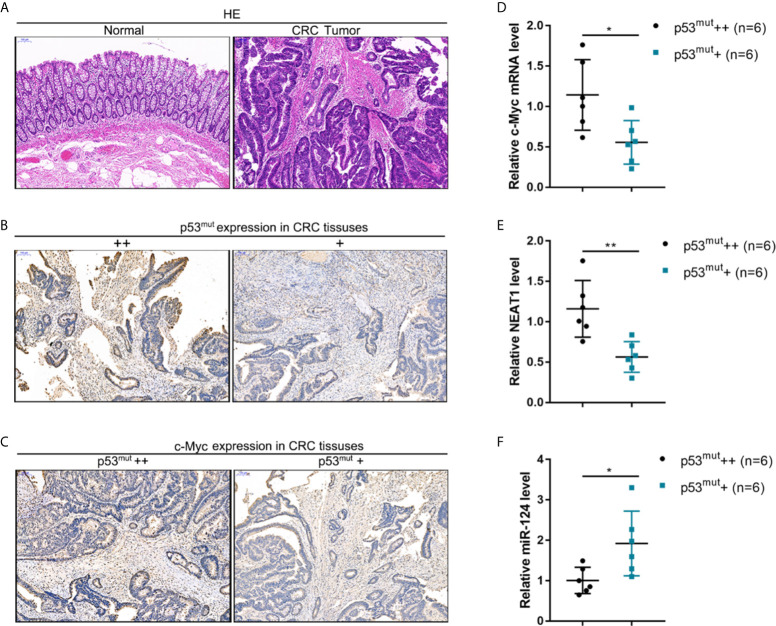
The expression of the c-Myc/NEAT1/miR-124 in p53-mutant tissues **(A)** CRC and adjacent non-cancerous tissue samples were collated, and the histopathological characteristics were examined by H&E staining. **(B)** By using an antibody specific to human p53 mutation, the p53 mutant in CRC tissue samples was identified by IHC staining. p53 mutant abundance was evaluated, and CRC tissues were divided into p53^mut^+ and p53^mut^++ groups accordingly. **(C)** C-Myc protein content was determined in p53^mut^+ and p53^mut^++ CRC tissues by IHC staining. **(D–F)** The expression levels of c-Myc, NEAT1, and miR-124 were determined in p53^mut^+ and p53^mut^++ CRC tissues by qRT-PCR. *P < 0.05, **P < 0.01 compared with p53^mut^+ group.

CRC and adjacent normal control tissues were collected, and c-Myc/NEAT1/miR-124 axis expression in p53-mutant CRC tissues was determined to verify the *in-vitro* findings further. The histopathological characteristics of human CRC and non-cancerous tissue samples were verified by H&E staining (**Figure 9A**). By using an antibody specific to human p53 mutation, the abundance of p53 mutant in CRC tissue samples was identified by IHC staining. As illustrated in **Figure 9B**, p53 mutant abundance was evaluated, and CRC tissues were divided into p53^mut^+ and p53^mut^++ groups accordingly. C-Myc protein content was determined in p53^mut^+ and p53^mut^++ CRC tissues for evaluating the proliferating status. As depicted in **Figure 9C**, C-Myc protein content was higher in p53^mut^++ CRC tissues. Next, the c-Myc/NEAT1/miR-124 axis was examined. In p53^mut^++ CRC tissue samples, c-Myc and NEAT1 expressions were shown to be higher, and miR-124 expression were shown to be lower compared with that in p53^mut^+ CRC tissues (**Figures 9D–F**). These *in vivo* findings are consistent with *in vitro* findings that p53^mut^ colon cancer cells show higher levels of c-Myc and NEAT1 but lower miR-124 levels.

## Discussion

This study confirmed that NEAT1 was downregulated within p53^wt^ HCT116 and RKO cells by PDT treatment. NEAT1 silencing enhanced the roles of PDT in the viability and apoptosis of cells in both these cell lines. Within the subcutaneously implanted tumor model, NEAT1 silencing promoted the suppressive roles of PDT in tumor growth. NEAT1 directly targeted miR-124; NEAT1 acted as a ceRNA, thereby competing with iASPP for miR-124 binding and counteracting miR-124-mediated repression on iASPP under PDT treatment. NEAT1 silencing enhanced, whereas miR-124 inhibition attenuated PDT effects on CRC cells; miR-124 inhibition significantly reversed the roles of NEAT1 silencing in PDT-treated CRC cells. Within tissues, miR-124 exhibited a negative correlation with NEAT1 and iASPP, respectively, and NEAT1 and iASPP positively correlated with each other. PDT downregulated c-Myc in CRC cells, and c-Myc activated the transcription of NEAT1 through the targeting of its promoter region. Under PDT treatment, cell proliferation and NEAT1 levels were lower in p53-overexpressed HCT116 cells compared with p53-deleted HCT116 cells. In comparison, the apoptosis rate was higher in p53-overexpressed HCT116 cells compared to p53-deleted HCT116 cells. Within p53^mut^ SW480 cells, PDT also failed to alter cell viability and apoptosis significantly. Under PDT treatment, the expression of c-Myc, NEAT1, and iASPP was higher, and the expression of miR-124 was lower in p53^mut^ SW480 cells. In c-Myc high-abundant CRC tissues, NEAT1 and iASPP were up-regulated, and miR-124 was downregulated. In p53 mutant high-abundant CRC tissues, c-Myc and NEAT1 were up-regulated, and miR-124 was downregulated.

The mechanisms of the acquisition of PDT resistance are complex, partially due to the numerous changes in genes and pathways that PDT treatment on cancer cells. By incubating oral squamous carcinoma Ca9-22 cells with four types of photosensitizers, Liu et al. (24) compared the genes differentially expressed between CRC cells with or without PDT treatment and found that LDLR, MMP10, and RHEBL1 had significant changes in all photosensitizer-mediated PDTs. As for CRC, Abrahamse et al. (25) found three upregulated and 20 downregulated genes within DLD-1 cells, while 16 upregulated and 22 downregulated genes within Caco-2 cells following a zinc (Zn) metal-based phthalocyanine (ZnPcSmix)-mediated PDT. Both pro-and anti-apoptotic genes were found among the upregulated genes. To identify more genes related to PDT functional mechanism, we also performed whole-transcriptome resequencing and found the dysregulation of 1096 lncRNAs within CRC HCT116 cells subjected to PDT treatment (12). In addition to lncRNA LIFR-AS1, which we already demonstrated to be involved in PDT functions on CRC cells (12), another lncRNA NEAT1 attracted our attention because of its downregulation in response to PDT treatment, as well as its oncogenic role in CRC (13, 26).

Previously, NEAT1 has been reported to increase within CRC (13–15) and serve as an oncogenic lncRNA through different mechanisms. Herein, the study found that NEAT1 was downregulated upon PDT treatment; moreover, the lncTar tool predicted that NEAT1 might target miR-124. In our previous study, it was demonstrated that the miR-124/iASPP axis exerted a critical effect upon PDT functions on CRC cells in a way related to the p53. When the p53 underwent mutation or deletion, PDT failed to alter CRC cell proliferation (11). Considering all the above findings, it was speculated that NEAT1 might also affect the PDT sensitivity of CRC cells. As expected, in p53^wt^ HCT116 and RKO cells, silencing NEAT1 enhanced PDT-mediated cell viability repression and PDT-stimulated cell apoptosis. Within a subcutaneously implanted tumor model in mice, silencing NEAT1 enhanced the inhibitory roles of PDT in tumor growth. *In vitro* and *in vivo* findings both support the speculation that NEAT1 participates in CRC cell sensitivity to PDT treatment. More importantly, experimental analyses then confirmed the online tool-predicted binding between NEAT1 and miR-124. Through competing with iASPP for miR-124 binding, NEAT1 counteracted miR-124–mediated repression on iASPP. In tissue samples, NEAT1 exhibited a negative correlation with miR-124 and a positive correlation with iASPP, suggesting the NEAT1/miR-124/iASPP axis in tissues.

P53 gene is an anti-tumor gene that frequently mutates in cancer (27, 28). The gain of function (GOF) effect caused by p53 mutation will increase the tolerance of cancer cells to PDT, including CRC (29, 30). In our previous study, our findings also evidenced that, when p53 mutant or deleted, the binding between p53 and miR-124 did not promote the expression of miR-124, and the expression of iASPP showed to be upregulated, eventually leading to enhanced viability of CRC cells in response to PDT treatment (11). Since NEAT1 is shown to affect PDT functions on CRC cells through the miR-124/iASPP axis, the role of the deletion or mutation of p53 in the process was subsequently investigated. Consistent with our previous study, in p53-deleted HCT116 cells or p53-mutant SW480 cells, PDT failed to cause significant changes in cancer cell proliferation; A significant NEAT1 downregulation by PDT, either in p53-deleted HCT116 cells or p53-mutant SW480 cells was observed. These findings indicate that PDT likely downregulates NEAT1 expression through other mediators.

The transcription factor is a protein that controls the transcription of the gene by targeting a specific DNA sequence (31, 32). Due to its action principle, PDT could alter the levels of a series of transcription factors related to oxidative stress or apoptosis, while these transcription factors, in turn, mediate cell survival or apoptosis following PDT (33). Herein, the present study determined c-Myc expression within CRC cells upon PDT and found that c-Myc was significantly downregulated. By both bioinformatics and experimental analyses, we confirmed the binding between c-Myc and the promoter region of NEAT1. Through the binding, c-Myc activated the transcription of NEAT1. Consistently, in p53^mut^ SW480 cells, PDT treatment failed to alter cell proliferation significantly but still significantly affected the expression of c-Myc, NEAT1, miR-124, iASPP. Within p53^wt^ RKO cells, the roles of PDT in c-Myc, NEAT1, miR-124, iASPP expression showed to be stronger than those on p53^mut^ SW480 cells, also suggesting the action of the miR-124/iASPP/p53 feedback loop on amplifying PDT functions.

**Figure 10:**
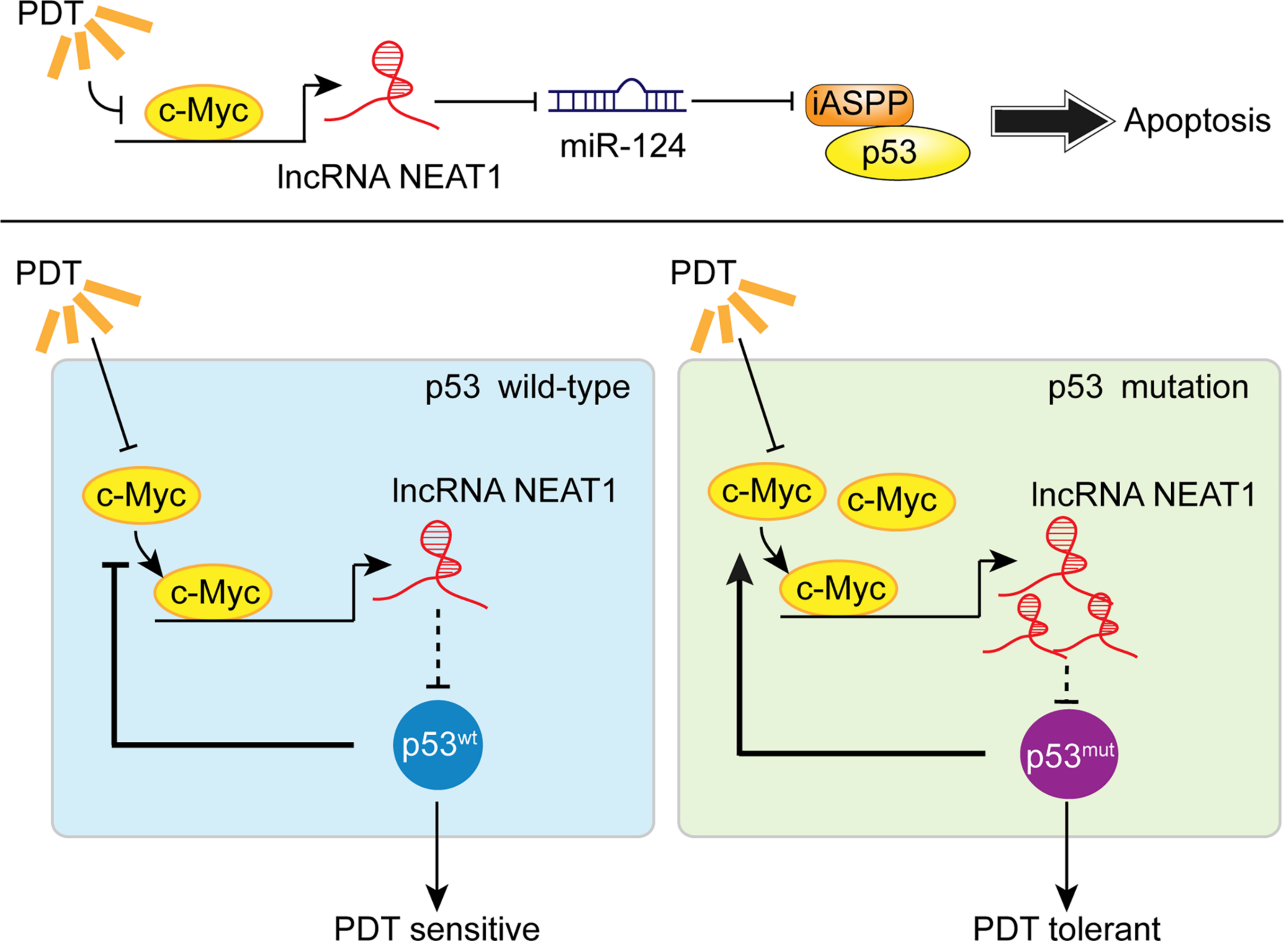
A schematic mechanism. In p53^wt^ CRC cells, PDT inhibits the c-Myc/NEAT1 axis and promotes the miR-124/iASPP/p53 feedback loop that amplifies PDT functions, inducing cancer cell apoptosis. In p53^mut^ CRC cells, the expression of the c-Myc/NEAT1 axis was up-regulated, and the miR-124/iASPP/p53 feedback loop could not be activated, leading to the failure in inducing apoptosis and, thus, PDT tolerance.

To further confirm these findings, we collected clinical samples and evaluated the p53 mutant abundance in CRC tissues by IHC staining. In p53 mutant high-abundant CRC tissues, c-Myc and NEAT1 expressions were shown to be higher, and miR-124 was shown to be lower. Similarly, in c-Myc high-abundant CRC tissues, the levels of NEAT1 and iASPP were higher, and miR-124 was lower. In conclusion, we demonstrated the critical role of c-Myc/NEAT1 axis in mediating CRC response to PDT treatment *via* the miR-124/iASPP/p53 feedback loop (**Figure 10**).

Regarding the limitation of the present study, other signaling pathways might be involved in PDT effects on CRC cells. Apoptosis (programmed cell death) is tightly controlled biochemically and genetically. It is divided into two pathways: the extrinsic or death receptor pathway and the intrinsic pathway (also known as the mitochondrial pathway). Both pathways are highly regulated and involve an extremely intricate play of several factors that activate either. Regarding PDT, the photosensitizer’s localization site indicates where the initial cellular damage will occur (34, 35). Kessel et al. (36) indicated that sub-cellular localization of the photosensitizer is a primary determinant of the cell death mechanism in PDT. Oxidative stress caused by photodynamic reactions or ROS production can directly disrupt the organelle membrane/s by peroxidation of membrane lipids (37). The JC-1 cationic dye demonstrates potential-dependent accumulation in the mitochondria, denoting mitochondrial depolarization and, subsequently, cell death (38). Besides, up-regulated death receptor 6 (DR6) may signal mitochondrial-induced cell death or induction of inflammation. Rupturing of lysosomes releases acidic lysosomal contents, such as cathepsins, consequently followed by activation of NAIP, which could precede inflammatory actions *via* NFkB-1 activation. The upregulation of *NFkB1* and the downregulation of *MCL1*, *BCL2L2*, and *BCL2L10* indicated unsuccessful evasion of apoptosis, DNA damage, and apoptosis (25). These previous findings suggest that the c-Myc/NEAT1 axis is one of the critical signaling pathways involved in CRC response to PDT treatment. Other signaling pathways should be investigated in the future.

## Data Availability Statement

The original contributions presented in the study are included in the article/**Supplementary Material**. Further inquiries can be directed to the corresponding author.

## Ethics Statement

This study was sanctioned by the ethics committee of Second Xiangya Hospital (2018(Yan149)). The patients/participants provided their written informed consent to participate in this study.

## Author Contributions

HY and KL designed the research and wrote the manuscript. SL and YK performed the majority of experiments. QJ collected and analyzed the clinical experimental data. DL and CL assisted with the animal experiments. YJ performed the figures and the statistical analyses. HZ provided the technical supports. All authors contributed to the article and approved the submitted version.

## Funding

This study was supported by the Natural Science Foundation of Hunan Province (2020JJ3054) and the Huxiang Youth Talents Supporting Program (No. 2020RC3062).

## Conflict of Interest

The authors declare that the research was conducted in the absence of any commercial or financial relationships that could be construed as a potential conflict of interest.

## Publisher’s Note

All claims expressed in this article are solely those of the authors and do not necessarily represent those of their affiliated organizations, or those of the publisher, the editors and the reviewers. Any product that may be evaluated in this article, or claim that may be made by its manufacturer, is not guaranteed or endorsed by the publisher.
