# Supplementary material for: A Novel Mechanism of the c-Myc/NEAT1 Axis Mediating Colorectal Cancer Cell Response to Photodynamic Therapy Treatment
Source: Front Oncol. 2021 Jul 28;11:652831. doi: 10.3389/fonc.2021.652831 (PMC8355738; doi:10.3389/fonc.2021.652831)
Supplement: Supplementary file 1 [file Table_1.docx]

Table S1 the primer sequence

| RT-PCR  NEAT1 | GCTCTTTTGTTCTTGCTTCGTTT | TCCCACCTCAGGGTCCTCTTA |
| --- | --- | --- |
| RT-PCR  MiR-124-3p | RT: GTCGTATCCAGTGCAGGGTCCGAGGTATTCGCACTGGATACGACGGCATT  F: GTCGCCTAAGGCACGCGGTG | GTGCAGGGTCCGAGGT |
| RT-PCR  U6 | CTCGCTTCGGCAGCACA | AACGCTTCACGAATTTGCGT |
| RT-PCR  GAPDH | CTCAGACGGCAGGTCAGGTCCACC | CCACCCATGGCAAATTCCATGGCA |
| RT-PCR  c-myc | GGCTCCTGGCAAAAGGTCA | CTGCGTAGTTGTGCTGATGT |
| RT-PCR  iASPP | GGCGGTGAAGGAGATGAAC | TGATGAGGAAATCCACGATAGAG |
| RT-PCR for CHIP  NEAT1 promoter | GGTCCTTTTTCATCCTCTACCTCAG | CGAATGTCTGGAGAGGACTTTGG |
| Agomir-NC | UUCUCCGAACGUGUCACGUTT | ACGUGACACGUUCGGAGAATT |
| Agomir-miR-124-3p | UAAGGCACGCGGUGAAUGCC | CAUUCACCGCGUGCCUUAUU |
| Antagomir-NC | CAGUACUUUUGUGUAGUACAA |  |
| Antagomir-miR-124-3p | GGCAUUCACCGCGUGCCUUA |  |
| Si-NC | UUCUCCGAACGUGUCACGUTT | ACGUGACACGUUCGGAGAATT |
| Si-C-Myc | GGAAGAAAUCGAUGUUGUUTT | AACAACAUCGAUUUCUUCCTT |
| pcDNA3.1-c-myc overexpression | ctagcgtttaaacttaagcttCTGGATTTTTTTCGGGTAGTGG | tgctggatatctgcagaattcTTACGCACAAGAGTTCCGTAGC |
| pcDNA3.1-p53^wt^ overexpression | ctagcgtttaaacttaagcttATGGAGGAGCCGCAGTCAG | tgctggatatctgcagaattcTCAGTCTGAGTCAGGCCCTTCT |
| Wt-lncNEAT1 | aattctaggcgatcgctcgagAACCAAGCTATTCCTAGGCCTGA | attttattgcggccagcggccgcCCTTCCCTGACCACTGCTGC |
| Mut-lncNEAT1 | TTGATtcgcggtgtGCACTCCAGCCTTGGCGA | TGCacaccgcgaATCAAAGCTTGCTGCAGCCT |
| Si-NEAT1 | GACAUAUAAUCAUGUAUAATT | UUAUACAUGAUUAUAUGUCTT |
| Si-NC | UUCUCCGAACGUGUCACGUTT | ACGUGACACGUUCGGAGAATT |
| Lv-sh-NEAT1 | GATCCGTGGTGTGTGTTGTGGAATCTCTCGAGAGATTCCACAACACACACCACTTTTTG | AATTCAAAAAGTGGTGTGTGTTGTGGAATCTCTCGAGAGATTCCACAACACACACCACG |
