# Supplementary material for: A Novel Mechanism of the c-Myc/NEAT1 Axis Mediating Colorectal Cancer Cell Response to Photodynamic Therapy Treatment
Source: Front Oncol. 2021 Jul 28;11:652831. doi: 10.3389/fonc.2021.652831 (PMC8355738; doi:10.3389/fonc.2021.652831)
Supplement: Supplementary file 2 [file Table_2.docx]

Table S2 Analysis of transcription factors binding to neat1 promoter by chromatin immunoprecipitation in CRC cells

| **ID** | **Antigen class** | **Antigen** | **Cell class** | **Cell** | **Num of peaks** |
| --- | --- | --- | --- | --- | --- |
| SRX359905 | TFs and others | MYC | Digestive tract | LoVo | 137 |
| SRX360576 | TFs and others | MYC | Digestive tract | GP5d | 3068 |
| SRX360590 | TFs and others | MYC | Digestive tract | GP5d | 602 |
| SRX361885 | TFs and others | MYC | Digestive tract | LoVo | 1005 |
| SRX361891 | TFs and others | MYC | Digestive tract | LoVo | 447 |
| SRX648243 | TFs and others | MYC | Digestive tract | LS-174T | 277 |
| SRX648246 | TFs and others | MYC | Digestive tract | LS-174T | 213 |
